# Research on the Improving Performance of Foam Concrete Applied to the Filling of Natural Gas Pipeline Cross-River Tunnel

**DOI:** 10.3390/ma15217461

**Published:** 2022-10-25

**Authors:** Xiaosong Ma, Chunbao Li, Haiyang Chen, Yongqi Wei, Yongmei Weng, Shen Li, Dalerjon Hojiboev

**Affiliations:** 1 Department of Civil Engineering, China University of Petroleum (East China), Qingdao 266580, China; 2 Key Laboratory of Advanced Civil Engineering Materials of Ministry of Education, Tongji University, Shanghai 201804, China; 3 School of Materials Science and Engineering, Tongji University, Shanghai 201804, China; 4 Construction Project Management Branch of China National Petroleum Pipeline Network Group Co., Ltd., Langfang 065001, China; 5 Mining-Metallurgical Institute of Tajikistan, Buston City 735730, Tajikistan

**Keywords:** foam concrete, tunnel filling material, mix ratio optimization, compressive strength, stability, dry density, ANSYS simulation

## Abstract

The shield tunnel is a common solution for natural gas pipelines crossing rivers. Consequently, the development of natural gas tunnel filling materials with excellent performance is crucial to the safe operation and maintenance of pipelines. The foam concrete offers a reasonable solution. Nevertheless, since its inherent compressive strength decreases almost proportionally with the decrease in density, obstacles remain concerning obtaining the high density and relatively low strength required for natural gas tunnel filling. Here, a synergistic optimization strategy was proposed involving the orthogonal test, univariate control, and comprehensive balance method. It involves modifying the type and proportion of cementitious matrix, in particular by incorporating fly ash and PVA fibers in the mix design, and synergetic determining the best mix ratio from the aspects of compressive strength, stability, and dry density. The obtained foam concrete has a compressive strength of 4.29 MPa (FC4) and a dry density of 1060.59 kg/m^3^ (A11), which meets the requirements of pipeline pressure and pipeline anti-floating. This study is applied to the Yangtze River shield crossing project of the Sino-Russian Eastern Gas Pipeline, and ANSYS was used to simulate the stress and deformation of the foam concrete. This work provides an efficient foam concrete optimization mix scheme, and supports the application of foam concrete in the filling of the long-distance cross-river natural gas tunnels.

## 1. Introduction

Natural gas resources are unevenly distributed worldwide, and therefore natural gas transmission pipelines are essential for the reallocation of these valuable resources. The construction of natural gas transmission pipelines often passes through a variety of complex terrains. A shield tunnel is the main solution for river crossing, the annular space between the tunnel wall and the natural gas pipeline is usually filled to protect the pipeline while facilitating operation and maintenance. Water, air, and controllable low-strength materials are the main tunnel-filling materials at present [1]. Thereinto, foam concrete has obvious advantages. It serves as a protective layer that can prevent direct contact between the pipe and water or air, and thus provides the pipes with good corrosion resistance and fireproof performance. Particularly, compared with water and air, foam concrete can also play a role in securing the pipe and strengthening the tunnel pipe sheet lining [2], and therefore it can improve the structural seismic performance of pipes. Consequently, foam concrete for tunnel filling has become one of the research hotspots.

Foam concrete is a lightweight porous material made by introducing air bubbles into the slurry mixed with cementitious materials, admixtures, and water [3]. Due to its adjustable density strength and ability to achieve self-leveling and self-compaction, foam concrete has been widely studied and applied in the past few decades. Most works are aimed at obtaining some special nonstructural functions such as thermal insulation, acoustic and sound insulation, vibration damping, and fireproof capability [4,5,6,7], etc. For example, Na et al. have studied the preparation process of foam concrete applied to mine filling roof [5]. Pasupathy et al. have prepared foam concrete with fine and regular pore size distribution using expanded perlite aggregate and used it as 3D printing material [8].

With the development and application of foam concrete in many fields, its shrinkage cracking and poor mechanical strength have become obstacles. Therefore, extensive exploration has been carried out around performance optimization [9,10,11,12,13]. Numerous studies aimed at increasing the typically low compressive strengths associated with low density by the introduction of fly ash and recycled fine particles, or polymer fibers [14,15,16,17,18]. For instance, Yuan et al. [17] found that a certain amount of fly ash addition could improve the dry-wet and freeze-thaw resistance of foam concrete, and the drying shrinkage is small. Tang et al. [18] reported that the addition of recycled PET fiber can improve the compressive strength and durability of foam concrete. The amount of fly ash in foam concrete is typically lower than 50% because high-volume fly ash could result in very low early-age strength due to its low reactivity [19,20]. Furthermore, the stability of foam concrete defined as the state of the mix at which the density ratio is closer to unity, depends on the consistency of the base mix [21,22]. Since the water-cement ratio and foaming agent properties affect the pore structure, the compressive strength and stability of foam concrete are significantly affected. [23,24,25]. For example, Falliano et al. [25] reported that foaming agent significantly affects the mechanical properties of foam concrete in the low-density range, and the combination of the foaming agent with a particular water-cement ratio is of crucial importance. Selija et al. [3] found that for foam concrete with different densities, there is an optimal water-cement ratio to obtain the optimal pore structure and mechanical strength. Some effective methods such as incorporating high elastic modulus polymer fibers are proposed to delay the development of cracks in foam concrete and improve its deformation resistance. Kudyakov et al. [26,27] found that a small number of asbestos fibers can reduce the drying shrinkage of foam concrete by more than 40%. Khan [28] studied the effect of polypropylene fibers on the splitting tensile strength and Poisson’s ratio of foam concrete and concluded that polypropylene fibers can substantially increase the tensile strength.

As discussed above, foam concrete shows good performance improvability. After filling the tunnel with foam concrete, the resistance to deformation of the natural gas pipeline is improved, and the compressive strength shall not be lower than the transmission pressure of the pipeline. Nevertheless, based on the design concept of the whole life cycle, the tunnels filled with foam concrete are exempt from routine maintenance, but emergency excavation for repair should be considered, so the strength of the foam concrete should be as low as possible when complying with the pressure in the natural gas pipeline. Additionally, considering the anti-floating demand of the pipeline, the density of foam concrete needs to be dense enough. Nevertheless, its inherent compressive strength decreases almost proportionally with the decrease in density [29]. Although many previous studies have made efforts to optimize the mechanical strength and dry density of foam concrete, it is still a great challenge for foam concrete to maintain a high dry density and obtain a relatively low mechanical strength to meet the filling requirements of the natural gas tunnel. Besides, considering the integrity of foam concrete when applied to fill long-distance tunnels, the instability of foam concrete in the fresh state [30,31] and the poor volumetric integrity due to shrinkage cracking [32] need to be noted.

Herein, in order to achieve the excellent filling effect, a synergistic optimization strategy of foam concrete mix ratio suitable for the filling of long-distance natural gas tunnels was proposed. Given the application of the Yangtze River shield crossing project of the Sino-Russian Eastern Gas Pipeline, by incorporating fly ash and PVA fibers in the mix design, the cementitious matrix was properly modified to achieve reasonable mechanical strength and density. The method of orthogonal test and controlling a single variable was used to synergetic determine the best ratio of water-cement ratio, fly ash substitution level, foaming agent dilution ratio, and superplasticizer from the aspects of compressive strength, dry density, and stability. Next, the PVA fibers were incorporated to prevent shrinkage and cracking of the bulk filler. The tested compressive strength of the optimized foam concrete was 4.29 MPa (FC4) and the dry density was 1060.59 kg/m^3^ (A11). It is important to verify the safety and reliability of the filling structure. The stress and deformation of foam concrete under the operating pressure of natural gas pipeline were simulated by ANSYS. The synergistic optimization of this investigation is conducive to obtaining the optimal mix ratio of foam concrete suitable for different filling projects, and foam concrete with optimized performance is a promising filling material for natural gas tunnels.

## 2. Materials and Methods

### 2.1. Raw Materials

Cement and fly ash were obtained from Qingdao Weidong Hongyuan Building Materials Co., Ltd. (Qingdao, China) Three types of silicate cement were studied in this study, and their basic properties are shown in Table 1. Grade II fly ash was used as supplementary cementitious material, and the chemical composition of fly ash is shown in Table 2. The foaming agent was HTW-1 compound foaming agent provided by Henan Huatai New Material Co., Ltd., (Nanyang, China) and the technical index is shown in Table 3. The superplasticizer was a polycarboxylic acid high-efficiency superplasticizer, and the mixing water was tap water. The PVA fiber was provided by Shanghai Chenqi Chemical Technology Co., Ltd., (Shanghai, China) and the technical index is shown in Table 4.

### 2.2. Specimen Preparation and Mix Design

**Figure 1:**
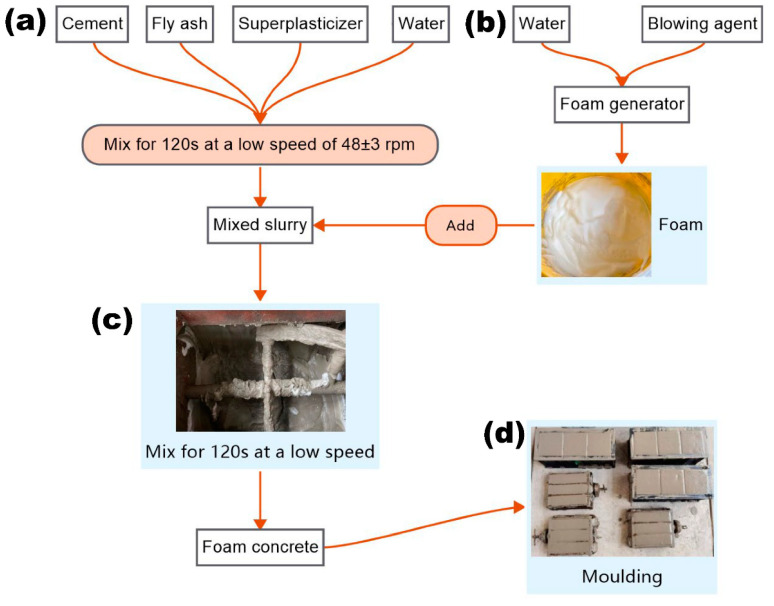
The preparation process of foam concrete.

Foam concrete was prepared by the pre-foaming method (Figure 1) as follows: cement and fly ash were added into the mixer and mixed at low speed (48 ± 3 rpm) for 2 min. Mixing water with polycarboxylate superplasticizer was then added and the mixture was mixed for 3–4 min to obtain a well-mixed slurry. The premade foams with a density of 68 kg/m^3^ were then added to the slurry to obtain the target density. The slurry was continued mixed at low speed for 2 min until the foam was well mixed with the slurry. The wet capacity of the foam concrete slurry in the fresh state was measured before casting, and finally, the specimens were poured into 40 mm × 40 mm × 160 mm and 100 mm × 100 mm × 100 mm molds brushed with lubricant and scraped calmly, and the specimens were demolded after 48 h and cured naturally until the specified age for performance testing.

In the orthogonal test, water-cement ratio (A), fly ash admixture (B), foamer dilution ratio (C) and water-reducing agent admixture (D) were selected as four factors, and four levels were set for each factor. Details of the orthogonal test are shown in Table 5.

### 2.3. Characterization

The 28 d compressive strength of foam concrete specimens was tested according to JC/T 266-2011 (Chinese industry standard). The specimen size was 100 mm × 100 mm × 100 mm. The test was performed on an electro-hydraulic universal testing machine (SHT4305, Sans, Shenzhen, China) with a loading rate of 2 kN/s, and the average value of three specimens was taken as the compressive strength.

The ratio of compressive-flexural strength is the ratio of compressive strength to flexural strength of the test block, which is used to reflect the anti-cracking performance of foam concrete. According to GB/T17671-2021 (Chinese national standard), the test block size was 40 mm × 40 mm × 160 mm. The test blocks were placed into the flexural jig and loaded on the upper surface of the flexural jig until the prismatic body fractured in the middle, with a loading rate of 50 N/s. The test blocks after fracture were taken for compressive strength testing with a loading rate of 2400 N/s ± 200 N/s, and the load was applied uniformly until the prismatic test blocks broke. The average values of three specimens were taken to calculate the compressive-fractural strength ratio.

The wet and dry densities of the foam concrete were measured in the fresh and hardened states, respectively. The fresh foam concrete slurry was poured into a measuring cup with volume V_0_, and the measuring cup was gently tapped to allow close packing of the slurry. The excessive slurry was scraped flat along the mouth of the measuring cup with a straightedge. The weight of the slurry (W_0_) was calculated after deducting the weight of the empty cup. The wet capacity was calculated as W_0_/V_0_. After being cured under natural conditions for 28 d, the hardened concretes were put into the drying oven and dried to constant weight, then taken out and placed in the room for cooling, and subsequently weighed to calculate their dry density.

## 3. Results and Discussion

### 3.1. Analysis of Extreme Differences in Orthogonal Tests

#### 3.1.1. Effect of Factors on Compressive Strength

**Figure 2:**
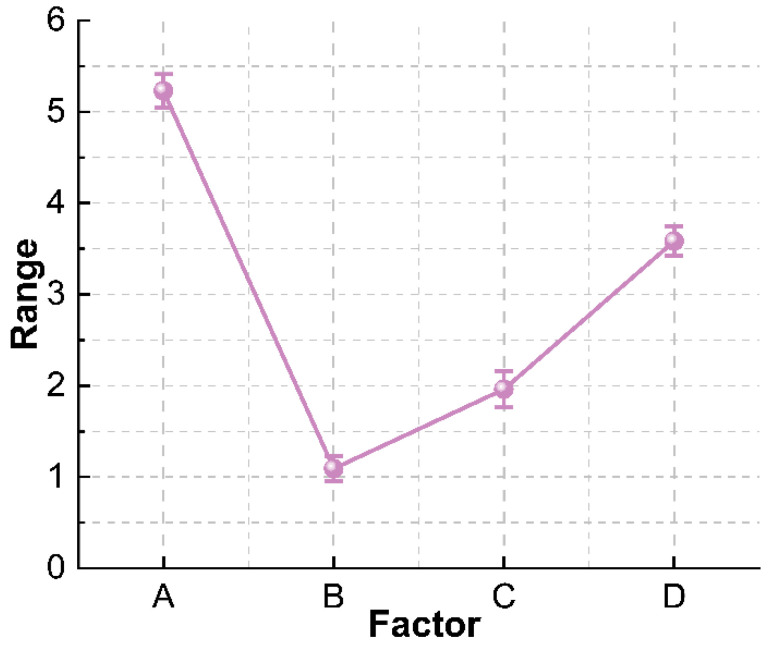
Analysis of the range of compressive strength of foam concrete.

Compressive strength is an important index that affects the application performance of foam concrete. Its low compressive strength is not conducive to providing sufficient support and protection for the pipeline. On the contrary, its high strength will make emergency repair difficult. The range analysis is carried out on the 28 d strength test results of the orthogonal test, and the compressive strength range analysis trend chart is made according to the range values of each factor, as shown in Figure 2. It can be seen that the influential factor in decreasing sequence on the 28 d compressive strength of foam concrete is water-cement ratio > water-reducing agent addition > foaming agent dilution ratio > fly ash addition. The water-cement ratio has the greatest influence on the compressive strength of foam concrete, and the addition of superplasticizer also has a great influence on the compressive strength, while the foaming agent dilution ratio and the fly ash addition have a small influence on the compressive strength. This is because the strength mainly comes from the hydration and hardening of the cementitious material [33]. 

**Figure 3:**
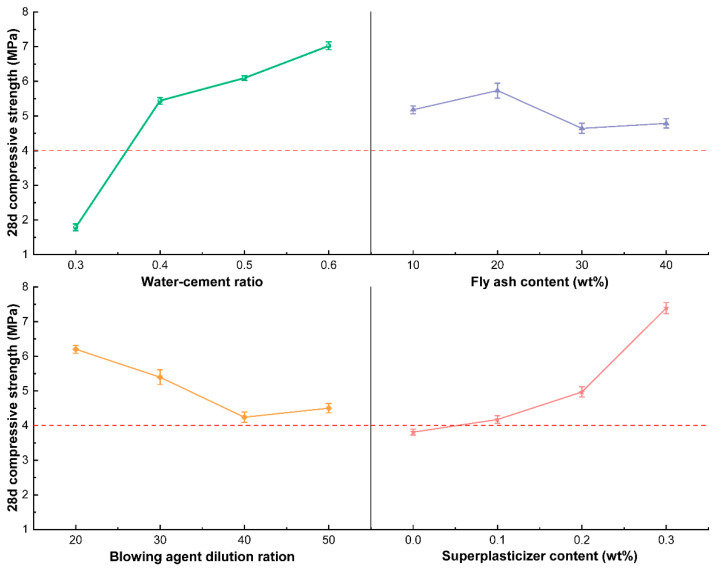
Effects of various factors on the compressive strength of foam concrete.

**Figure 4:**
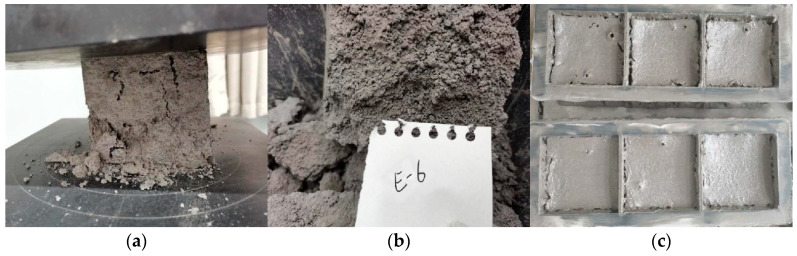
Representative results of compressive strength tests: (**a**) The shape of the test block damaged by compression; (**b**) The failure section of the specimen with a water-cement ratio of 0.3; (**c**) The foams are unstable and break when the blowing agent is diluted 50 times.

In order to directly reflect the impact of each factor level change on the 28 d compressive strength, the trend chart of factors and indicators is drawn as shown in Figure 3. It can be seen that the compressive strength of foam concrete is significantly increased when the water-cement ratio is increased from 0.3 to 0.6. On the one hand, the water-cement ratio directly affects the hydration process of the cementitious material, which in turn affects the compressive strength of the foam concrete. The lower water-cement ratio is not conducive to the hydration and hardening of the cementitious material, resulting in a large number of unhydrated cementitious material agglomerates inside the foam concrete (Figure 4b). On the other hand, in a slurry with a low water-cement ratio, the foams are difficult to mix uniformly with the slurry, and the foams tend to break down into a dilute slurry, leading to large pores and structural defects in the hardened cement and thus a lower compressive strength. Within an addition of less than 0.3%, increasing the addition of superplasticizer increases the compressive strength of foam concrete significantly. This is mainly because the addition of a superplasticizer improves the dispersion of the cementing material. At the same time, the setting and hardening speed of foam concrete is accelerated under the action of the water-reducing agent. Consequently, the connected pores are reduced, and the pores are more uniformly distributed in foam concrete. With the increase of the dilution ratio of the foaming agent, the compressive strength of foam concrete shows a trend of gradual decrease, which is mainly related to the stability of the foam. When the dilution ratio of the foaming agent is too high, the foam produced becomes less stable and easy to break (Figure 4c). The fly ash has a much slighter effect on compressive strength. The fineness of fly ash is lower than that of cement, and therefore fly ash can be evenly dispersed in the slurry. The hardened concrete fills the capillary pores and improves the pore wall structure, giving the concrete higher compressive strength. However, fly ash is mainly in the form of solid microbeads with smooth surfaces, which plays the role of roller ball lubrication, and hence excessive addition of fly ash will lead to the increase of pore size and connected pores, and the compressive strength will be reduced correspondingly. Combined with the above analysis, the influence of various factors on the strength mainly by affecting the hardening of cementitious materials and the internal pore structure. The foam concretes with uniform distribution of pore structure, concentrated pore size distribution, and a smaller number of connected pores and large pores show better compressive properties [34,35,36]. The best level combination for the target 28-day compressive strength is selected as A2B3C3D2.

#### 3.1.2. Effect of Factors on Concrete Stability

**Figure 5:**
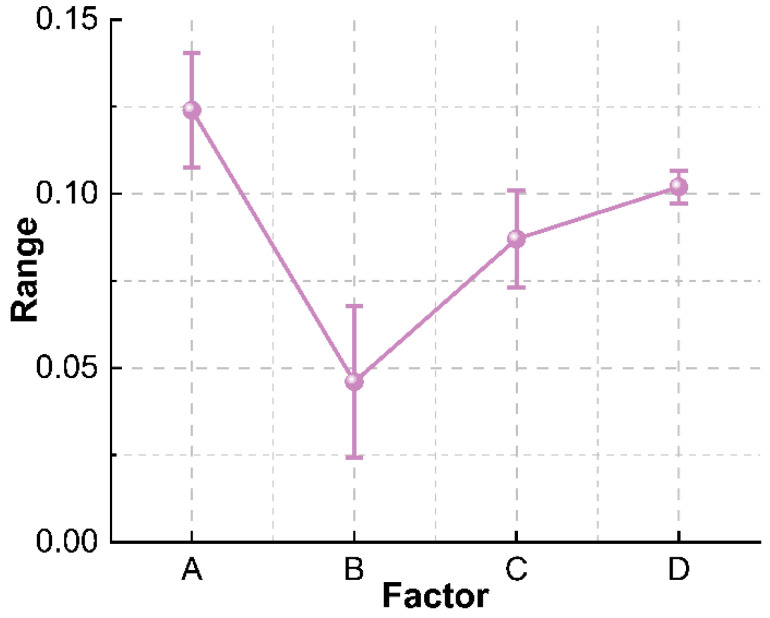
Analysis of the range of stability of foam concrete.

Due to the small inner diameter of the natural gas tunnel and the arrangement of gas pipelines inside it, the slurry should be self-leveling, and therefore self-compacting foam concrete should be used. The self-compacting foam concrete is required to have high stability, which can be characterized by the foaming volume ratio (slurry wet density/dry density). The stability of the foam concrete is better if the foaming volume ratio is close to 1 and there is no segregation and water secretion during the mixing and setting of the slurry [37]. The range analysis is carried out on the stability test results of the orthogonal test, and the stability range analysis trend chart is made according to the range values of each factor, as shown in Figure 5. The results show that the extent of influence of each factor on the stability of foam concrete is water-cement ratio > superplasticizer addition > foaming agent dilution ratio > fly ash addition. The water-cement ratio has the greatest influence on the stability, and the superplasticizer addition and the foaming agent dilution ratio also have a great influence. This result is consistent with the 28 d compressive strength analysis result.

**Figure 6:**
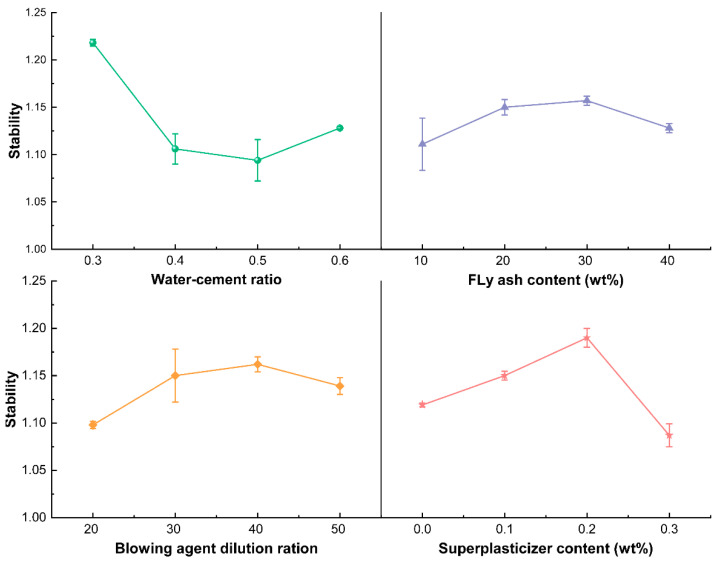
Effects of various factors on the stability of foam concrete.

**Figure 7:**
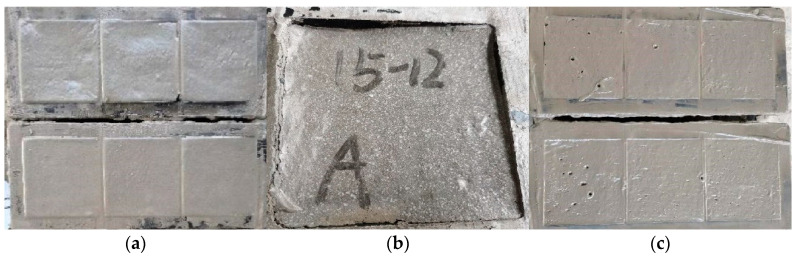
(**a**) The foam concrete with the best ratio has good stability; (**b**) The foam concrete slurry with water cement ratio of 0.6 segregates and bleeding leads to collapse; (**c**) The water-reducing agent is 0.2, and the foam concrete bubbles merge and float up.

The influences of the change of factors on the stability of foam concrete are further analyzed, as shown in Figure 6. It can be seen that the influences of factors on stability are consistent with their influences on compressive strength. Foam concrete with good stability also has better strength performance, which is in line with the basic law of porous materials [38]. With the increase of the water-cement ratio, the stability of foam concrete increases first and then decreases, and the stability of foam concrete is best when the water-cement ratio is 0.5. The water-cement ratio mainly affects the stability of foam by influencing the compatibility of foam and slurry in the mixing process. When the water-cement ratio is too large, the excess free water will form mobile channels inside the slurry, leading to bleeding (Figure 7b). When the addition of superplasticizer is too low, foam concrete stability will be reduced. In this condition, the stability of foam will be impaired, resulting in the continuous fusion of small bubbles in the slurry to produce large bubbles that eventually float up and break down (Figure 7c). The stability of foam concrete begins to improve significantly when the amount of superplasticizer is greater than 0.2%. At this time, the benefits of the superplasticizer on the stability of foam concrete begin to be significant. It improves the compatibility of the mixed slurry, making the foams uniformly distributed in the slurry in a stable manner. The foaming agent is the key factor to determine the quality of foam. The increase in the dilution ratio of the foaming agent will lead to a reduction in the thickness and integrity of the resulting foam film, making the produced foams unstable, which in turn leads to poor stability of foam concrete. The stability of foam concrete decreases after the addition of fly ash. This is due to the fact that fly ash consists of a large number of solid microbeads with smooth surfaces and a small number of hollow microbeads, which play the role of lubrication and thus lead to an increase in internal connecting pores. With the best stability as the goal, A3B1C1D4 is selected as the best combination.

#### 3.1.3. Standard Mixing Proportion

According to the target compressive strength and stability of foam concrete, the optimal level combinations of factors are different. Based on the results of compressive strength and stability extreme difference analysis, the comprehensive balance method was used to compare the four factors respectively, the analysis process is shown in Table 6. The level combination for the target compressive strength and better stability was selected as the standard mixing proportion of foam concrete. The factor levels and performance test results are shown in Table 7.

### 3.2. Properties Improvement Based on Practical Application

Compressive strength is one of the crucial properties when foam concrete is applied to fill natural gas tunnels. Foam concrete with high compressive strength provide greater restraint on a natural gas pipeline, and the filled tunnel can provide better bearing capacity to protect the pipeline. However, too high a strength can cause difficulties in emergency tunnel excavation and repair. From the test results of the standard mixing proportion, it can be seen that the compressive strength of the foam concrete is large than the target compressive strength FC4, and further optimization is thus needed to make it meet the target requirements. At the same time, the dry density is an important performance index in the optimization process. The density of optimized foam concrete should reach the grade requirement of A11 to prevent a pipe from floating.

**Figure 8:**
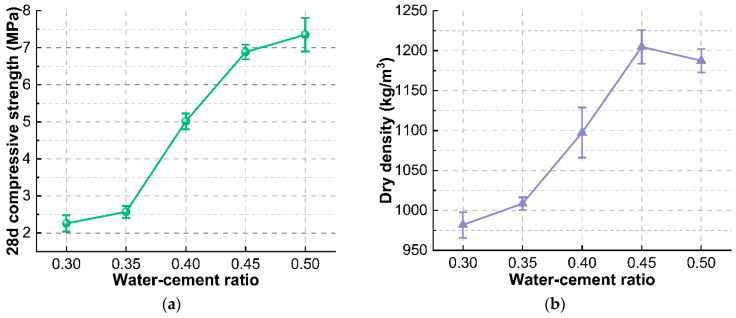
Effect of water-cement ratio on (**a**) 28 d compressive strength and (**b**) dry density of foam concrete.

From the results of the multi-factor test, it is clear that the water-cement ratio is the most important factor affecting the compressive strength of foam concrete. In order to optimize the compressive strength to meet the target requirements, further single-factor optimization of the water-cement ratio was carried out. It can be seen that the compressive strength of foam concrete changes sensitively when the water-cement ratio is varied in the range of 0.35 to 0.45 (Figure 8a), and the dry density is changed together with the compressive strength (Figure 8b). The increase in the water-cement ratio can significantly improve the dry density of foam concrete within a certain range, and the dry density reaches the maximum when the water-cement ratio is 0.45. Further increase in the water-cement ratio will make the foam concrete easy to stratify and bleed during mixing and forming, resulting in the increase of the ratio of internal connecting pores and the decrease of dry density of the product. Reducing the water-cement ratio can effectively reduce the compressive strength of foam concrete, but also brings a great loss in dry density. The optimized water-cement ratio is 0.35, which is determined by requirements on the comprehensive strength and density.

**Figure 9:**
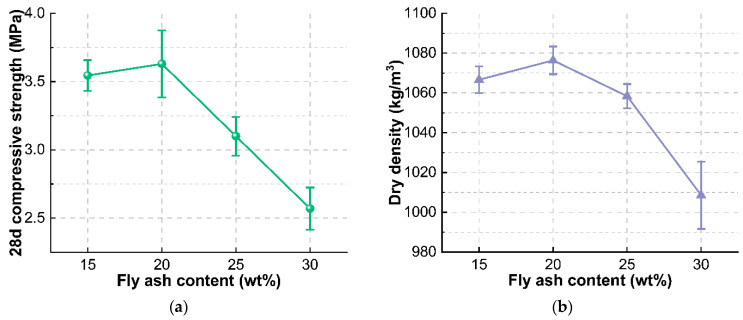
Effect of fly ash content on (**a**) 28 d compressive strength and (**b**) dry density of foamed concrete.

**Figure 10:**
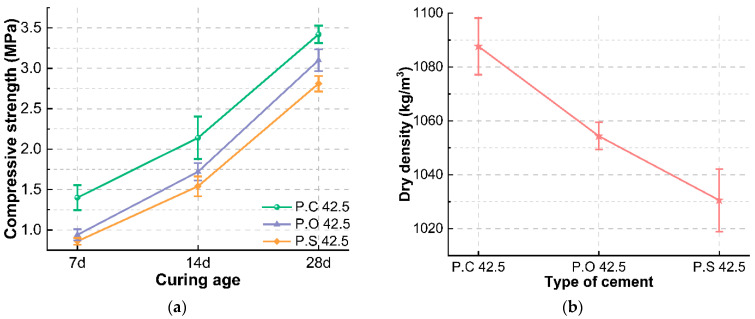
Effect of cement types on (**a**) compressive strength at different ages and (**b**) dry density of foam concrete.

The compressive strength of the foam concrete obtained after water-cement ratio optimization is 2.67 MPa and the dry density is 1028.59 kg/m^3^, both of which are slightly lower than the target values. The type and usage of cementitious materials are the key factors affecting the performance of foam concrete. Fly ash, as supplementary cementitious material with a smaller particle size than cement, can regulate the particle gradation of cementitious materials and optimize carbon emission at the same time. As seen from Figure 9, it can be seen that the dry density of foam concrete firstly increases and then decreases with the increase of fly ash substitute level, which is consistent with the change of compressive strength. The fly ash can evenly disperse in the slurry and fill into the pores, so that the density increases. When the addition of fly ash exceeds the optimum addition, the ball lubrication effect leads to the increase of the pore size and the connected pores, and a decrease of compactness. Therefore, the dry density is increased to meet the target performance requirements by reducing the fly ash addition to 25%. In addition, cement is the main cementitious material of foam concrete, and the suitable type of cement is not only beneficial to performance improvement but also brings a better economy. The effect of cement type on the performance of foam concrete was studied on the basis of optimized mixing proportion. The density and strength of foam concrete prepared by different types of cement are shown in Figure 10. It shows that foam concrete prepared by P.C 42.5 can obtain higher early strength, which is beneficial to reduce foam rupture and increase the stability of foam concrete, and the 28 d compressive strength and density of the prepared foam concrete are higher than those of concrete made of P.O 42.5 and P.S 42.5 cement. Based on the above analysis, the optimum replacement rate of fly ash is determined to be 25% and the P.C 42.5 cement is selected. Under these conditions, the compressive strength and dry density of the prepared foam concrete are increased to 3.42 MPa and 1087.64 kg/m^3^, respectively, which are close to the target values. The prepared foam concrete has a low cost.

**Figure 11:**
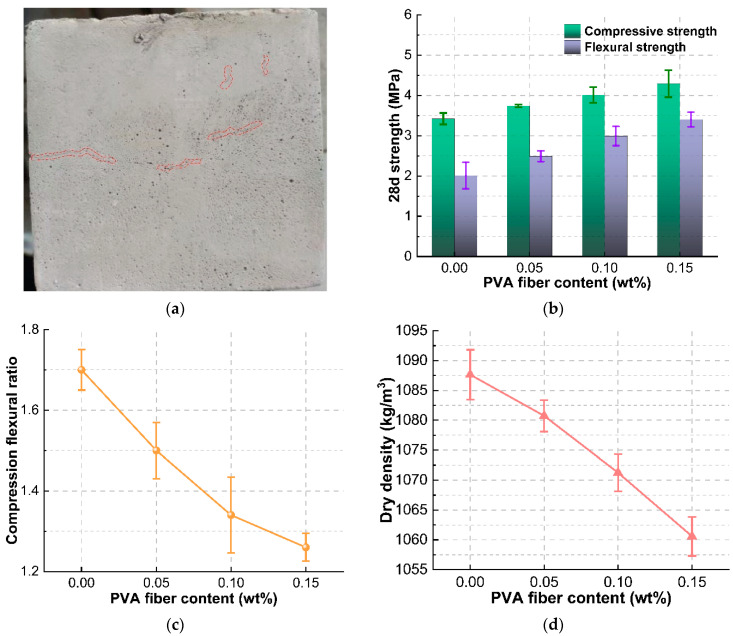
(**a**) Micro cracks on the surface of foamed concrete specimens; (**b**) Effect of PVA fiber content on compressive and flexural strength; (**c**) Effect of PVA fiber content on compression flexural ratio; (**d**) Effect of PVA fiber content on dry density.

The foam concrete produced after optimization shows fine cracks on the surface (Figure 11a), indicating the foam concrete has poor cracking resistance. The PVA fibers have good affinity with cement-based cementitious materials [39], and in order to ensure the integrity of foam concrete, the PVA fibers with high elastic modulus are incorporated to improve the cracking resistance. The compressive-flexural strength ratio is an important index to characterize the flexibility of foam concrete, which can well reflect the cracking resistance of foam concrete. The effect of PVA fiber addition on the flexural properties of foam concrete is shown in Figure 11. It can be seen that fiber incorporation is beneficial to improve the flexural strength and compressive strength of foam concrete. With the increase of fiber addition, the compressive-flexural strength ratio of foam concrete decreases, and the cracking resistance of foam concrete is improved. The compressive-flexural strength ratio is the smallest when the addition of PVA fiber is 0.15%. This is because, in the mixing process, the PVA fiber flakes become a large number of filaments that are irregularly distributed in the foam concrete slurry to play the role of “weak reinforcement”. The fiber filaments alleviate the shrinkage of the foam concrete to a certain extent in the drying and hardening process. The PVA fiber can also delay the development of foam concrete cracking and effectively inhibit the occurrence of small cracks in the hardening process of fiber foam concrete. Based on the experimental results, the optimal content of PVA fiber is selected as 0.15%, at this time the compression flexural ratio is the smallest, and the foam concrete after forming had better crack resistance.

Therefore, based on the comprehensive analysis above, the optimized mixing proportion of foam concrete is shown in Table 8. The compressive strength of foam concrete is 4.29 MPa (FC4) and the dry density is 1060.59 kg/m^3^ (A11), which meets the requirements of the target performance. Meanwhile, the strength of foam concrete develops very fast and has good anti-cracking performance, which can be applied to long-distance tunnel-filling applications.

### 3.3. ANSYS Simulation during Operation Phase

The Yangtze River crossing project of the China–Russia East Line is characterized by long crossing distances and a large diameter natural gas pipeline arranged in the tunnel, which makes the foam concrete have a larger filling volume and complex structure. In order to ensure the safety and stability of the tunnel as a whole during the operation phase and avoid the damage of foam concrete due to excessive stress or deformation, the ANSYS software is used to simulate the actual working conditions of foam concrete during the operation phase of the tunnel, so as to ensure that the foam concrete with optimized mixing proportion meets the requirements of engineering applications. 

#### 3.3.1. Uniaxial Compressive Constitutive Model of Foam Concrete

**Figure 12:**
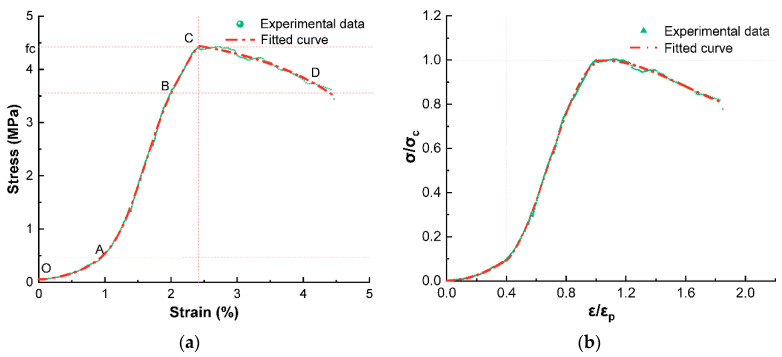
(**a**) The stress-strain curve of foam concrete with optimized mix ratio. (**b**) Dimensionless stress-strain curve of foam concrete with optimized mix ratio.

The constitutive relationship of foam concrete under uniaxial compression is the main basis for the selection of each parameter index for finite element simulation, which can fully reflect the basic characteristics of foam concrete such as strength and elastic-plastic deformation [40,41]. Foam concrete is a nonlinear material similar to ordinary concrete [31], and uniaxial compression experiments were conducted on foam concrete by short-term loading, i.e., the load was monotonically increased from zero until the foam concrete specimens were broken. The stress-strain curve of the foam concrete with this ratio was obtained as shown in Figure 12a. In order to better describe the stress-strain variation law at each stage, the stress-strain test results were standardized to obtain a typical stress-strain relationship curve as shown in Figure 12b.

The stress-strain curve to a certain extent reflects the changes in the internal structure during the uniaxial compression damage of foam concrete. Combined with Figure 12a, it can be seen that the process of uniaxial compression damage of foam concrete is divided into four stages. When the stress is less than 0.1 fc (OA), the stress grows more slowly with the increase of foam concrete deformation, and the deformation of foam concrete in this stage is mainly due to the closure of the internal pore structure of foam concrete under the action of load. As the load continues to be applied (AB), the rate of stress increase is much larger than the rate of strain increase. The linear stress-strain curve shows that elastic deformation occurs in this period. The slope of AB is the modulus of elasticity (3000 MPa) of foam concrete, which is about 0.1 times that of ordinary concrete. The critical stress at point B can be seen as the long-term compressive force to which the foam concrete is subjected. When the stress value approaches the peak stress fc (BC), the cracks further develop in a stable manner. After reaching the ultimate stress (CD), the strain continues to increase and cracks continue to develop in terms of number and width until complete destruction. It is worth noting that the stress decreases more slowly in this process and the values show certain fluctuations, which may be due to the poor distribution of the internal pore in the foam concrete, resulting in stress concentration in multiple locations.

Taking the peak compressive strength as the dividing point, the typical stress-strain curve is divided into ascending and descending segments. According to the shapes of the rising and falling sections, the curves are fitted by segments, with the rising section being fitted in two parts and the falling section being fitted by a polynomial cubic. The uniaxial compressive stress-strain constitutive equation of optimized ratio foam concrete is shown in Equation (1).

(1) $$y=\left\{\begin{array}{cc} 0.013x+0{.561x}^{2} & \;0\;\leq \;x\;\leq \;0.4\\ -6.02x+11{.735x}^{2\;}-\;5{.717x}^{3} & \;0.4\;\leq \;x\;\leq \;1.0\\ 2.477x\;-\;1{.758x}^{2}+0{.368x}^{3} & \;x\;\geq \;1.0 \end{array}\right\}$$

#### 3.3.2. Operation Simulation of Foam Concrete

**Figure 13:**
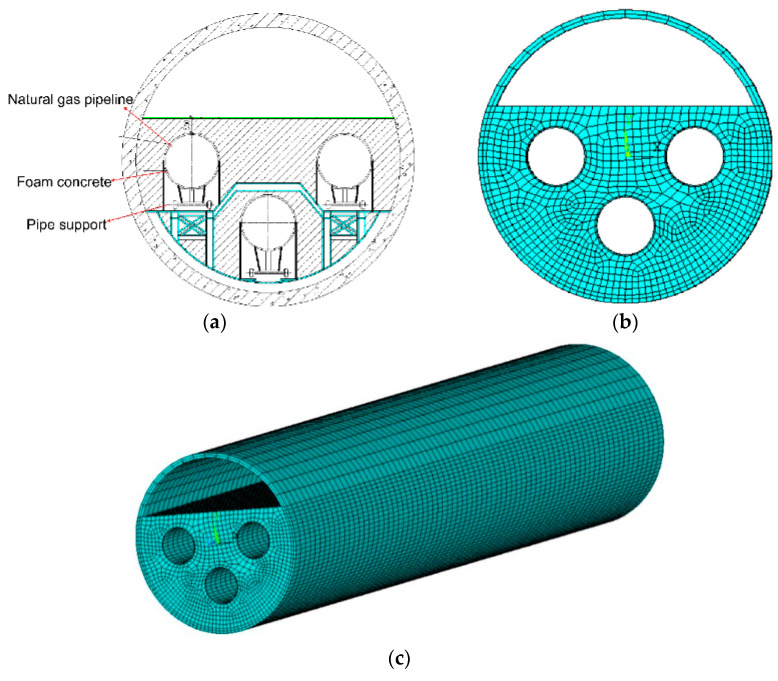
(**a**) Schematic diagram of shield tunnel section; (**b**,**c**) Finite element model of foam concrete filled tunnel.

The ANSYS software was used to simulate the working conditions of the foam concrete filler during operation, and foam concrete in a horizontal section of the tunnel was selected for analysis. The shield tunnel lining concrete, natural gas pipe, and foam concrete are all isotropic linear elastic materials, and the material properties are assigned according to the material properties as shown in Table 9. The model parameters of the foam concrete-filled tunnel are shown in Table 10, and the gas pipeline tunnel model is established by combining the shield tunnel section layout (Figure 13a). Different methods were used to mesh the tunnel lining concrete ring, natural gas pipeline, and foam concrete, as shown in Figure 13b,c. The foam concrete filler has a certain self-stability after hardening, which has a certain restraint effect on the gas pipe. There is also certain bond strength between the pipe and the foam concrete. This model is based on the simplified relationship without considering the bond-slip relationship between the axial pipe and foam concrete. The pipe and foam concrete are assumed to contact each other directly, and the liner outside is set as fully restrained. The design operating pressure of the natural gas transmission pipeline is 10 MPa. The ANSYS finite element calculation is thus carried out for the foam concrete filled in the annular space.

**Figure 14:**
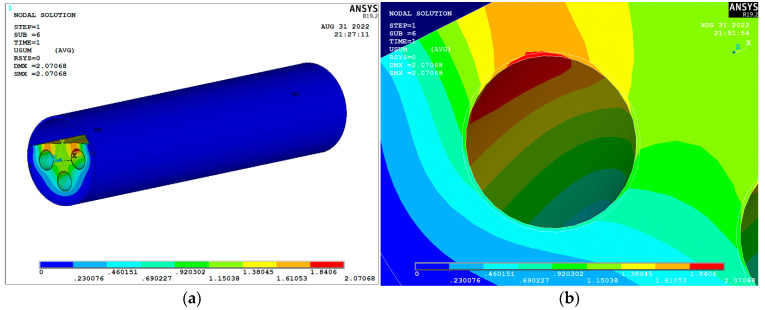
Deformation cloud chart under operating pressure: (**a**) Natural gas transmission tunnel; (**b**) Gas pipeline.

**Figure 15:**
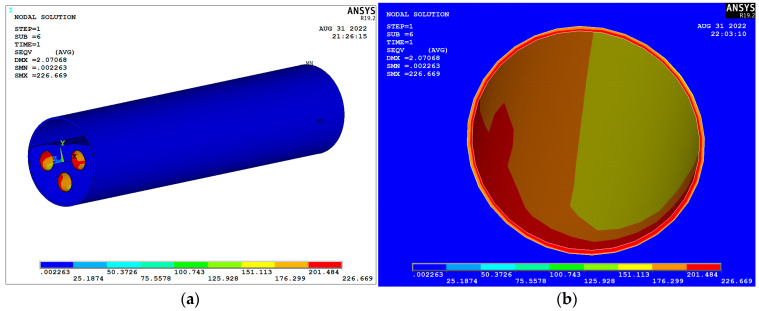
Stress cloud chart under operating pressure: (**a**) Natural gas transmission tunnel; (**b**) Gas pipeline.

The overall plastic strain and stress in the foam concrete filled in the natural gas transmission tunnel during operation are shown in Figure 14 and Figure 15. From Figure 14, it can be seen that the maximum deformation in the natural gas pipeline under the design transmission pressure is 2.07 mm, and the maximum value of displacement of foam concrete under the action of the pipeline is 1.61 mm, which occurs at the thinnest location of foam concrete filling, i.e., which is at above the natural gas pipeline. The overall deformation of the natural gas pipe and foam concrete filling body is small, and the foam concrete plays a good restraining role for the natural gas pipe. Figure 15 shows that the maximum stress of foam concrete does not exceed 3.5 MPa under the action of the pipeline, and the compressive strength of foam concrete with optimized mixing proportion is 4 MPa, so the plastic damage of foam concrete will not take place under the design working condition. The above results show that the optimized foam concrete can better meet the performance requirements for filling the annular space between the river crossing tunnel and the inset natural gas pipeline.

## 4. Conclusions

In this work, the optimum mix ratio of foam concrete suitable for filling long-distance cross-river natural gas tunnels was studied. The orthogonal test and single factor test were carried out to discuss the effects of water-cement ratio, water-reducing agent addition, foam dilution ratio, and fly ash addition on stability and 28d compressive strength. The requirements of dry density and crack resistance have been considered, including three types of cement and PVA fiber. The following conclusions can be drawn as major outcomes of the present study:

(1) The significance of the effects of various factors on the compressive strength and stability of foam concrete is consistent. The factors, in the decreasing sequence in terms of their significance, are water-cement ratio, water-reducing agent addition, foam dilution ratio, and fly ash addition. The increase in water-cement ratio and water-reducing agent can significantly improve the compressive strength and stability of foam concrete within a certain range. This is mainly because the two factors influence the hydration of cementitious materials and the stability of foams in slurry. There is an optimal dilution ratio of a foaming agent to obtain the best foam stability, which is conducive to improving the compressive strength and stability. The appropriate amount of fly ash can improve the pore structure and thus increases the compressive strength and stability of foam concrete.
(2) The dry density increases almost proportionally with compressive strength, and the addition of PVA fiber can improve compressive strength and flexural strength, and significantly improve the anti-cracking performance of foam concrete. Based on the performance requirements on long-distance river-crossing tunnel, an optimal mixing proportion of foam concrete is proposed as: water-cement ratio of 0.35, fly ash admixture of 25%, 40 times dilution of foaming agent, 0.1% of high-efficiency superplasticizer, and 0.15% of PVA fiber. The compressive strength of the optimized foam concrete is 4.29 MPa (FC4) and the dry density is 1060.59 kg/m^3^ (A11). The ANSYS simulation results show that the maximum deformation of foam concrete during the operation of the natural gas pipeline is 1.61 mm, and the maximum stress does not exceed 3.5 MPa. Plastic damage of foam concrete will not take place during operation. The designed foam concrete meets the safe operation demand for natural gas pipeline tunnel filling projects.
(3) The present research work has been only focused on compressive strength, dry density, and stability. Additional properties important in long-distance gas tunnel filling applications such as fluidity, corrosivity, and pipeline adhesion will be explored in a forthcoming study. Moreover, further research will be aimed at high-performance foaming agents and recycled admixtures to make this research environmentally friendly.

## Figures and Tables

**Table 1:** Basic properties of cement.

Cement Grade	Specific Surface Area (kg/m^2^)	Setting Time (min)		Flexural Strength (MPa)		Compressive Strength (MPa)	
		Initial	Final	3 d	7 d	3 d	7 d
P.C 42.5	325	169	217	5.3	8.9	27.2	43.9
P.O 42.5	321	125	225	6.6	9.2	30.0	55.8
P.S 42.5	307	181	230	4.6	7.9	21.7	41.5

**Table 2:** The chemical component of fly ash.

Constituents	SiO_2_	Al_2_O_3_	CaO	Fe_2_O_3_	K_2_O	TiO_2_	Loss on Ignition
wt%	54.94	34.86	2.63	2.52	1.76	1.25	2.04

**Table 3:** Characteristics of compound foam agent.

Label	Appearance	PH	Foam Expansion Ratio	Dilution Ratio	Bleeding Rate for 1 h (mL)	Sedimentation for 1 h (mm)
HTW-1	Light yellow liquid	8.25 ± 0.75	28	40	35	8.3

**Table 4:** Basic properties of PVA fiber.

Colour	Density (g/m^3^)	Length (mm)	Diameter (μm)	Tensile Strength (MPa)	Elastic Modulus (GPa)	Elongation (%)
White	1.29	3–12	31.0	1500	35.0	8.0

**Table 5:** Orthogonal test factors and levels of foam concrete.

Factor	Level			
(A) Water-cement ratio	(1) 0.3	(2) 0.4	(3) 0.5	(4) 0.6
(B) Fly ash content	(1) 10%	(2) 20%	(3) 30%	(4) 40%

**Table 6:** Comprehensive balance analysis table of factors.

Level	28 d Compressive Strength (MPa)	Stability	Favorable	Unfavorable	Result
A2	5.440	1.106	11.99%	10.85%	A2
A3	6.092	1.094	10.97%	10.70%	
B3	4.643	1.157	23.45%	3.98%	B3
B1	5.732	1.111	4.14%	19.00%	
C3	4.242	1.162	6.13%	5.51%	C3
C1	4.502	1.098	5.83%	5.78%	
D2	4.175	1.150	77.01%	5.48%	D2
D4	7.390	1.087	5.80%	43.50%	

**Table 7:** Foundation mix ratio and properties of foam concrete.

W/C	FA	BA	SP	28 d Compressive Strength (MPa)	Dry Density (kg/m^3^)	Stability
0.4	30%	40	0.1%	5.015	1097.58	1.112

Note: W/C represents the water-cement ratio; FA represents the fly ash content; BA represents the blowing agent dilution ratio; SP represents the superplasticizer content.

**Table 8:** Optimal mix ratio of foam concrete based on engineering properties.

W/C	FA	BA	SP	Type of Cement	PVA Fiber Content
0.35	25%	40	0.1%	P.C 42.5	0.15%

Note: W/C represents the water-cement ratio; FA represents the fly ash content; BA represents the blowing agent dilution ratio; SP represents the superplasticizer content.

**Table 9:** Material parameters of shield tunnel for natural gas pipeline.

Part	Density (kg/mm^3^)	Elastic Modulus (MPa)	Poisson’s Ratio	Shear Modulus (MPa)
Shield tunnel lining concrete ring	2.50 × 10^−6^	3 × 10^4^	0.2	1.2 × 10^4^
Natural gas pipeline	7.85 × 10^−6^	2.06 × 10^5^	0.3	7.9 × 10^4^
Foam concrete	1.06 × 10^−6^	3 × 10^3^	0.2	1.2 × 10^3^

**Table 10:** Model parameters for foam concrete-filled tunnels.

Part	Inner Diameter (mm)	Outer Diameter (mm)	Length (mm)
Shield tunnel	6800	7600	30,000
Natural gas pipeline	1422	1486	30,000

## Data Availability

The data used to support the findings of this study are available from the corresponding author upon request.
